# Solitary Ocular Plasmacytoma: Exploring a Rare Extramedullary Tumor

**DOI:** 10.7759/cureus.77846

**Published:** 2025-01-22

**Authors:** Andrea González-Espinoza, Melissa Flores-Marín, Abelardo A Rodriguez-Reyes, Alexis Zambrano-Zambrano

**Affiliations:** 1 Department of Ophthalmology, Hospital Regional "Lic. Adolfo López Mateos" ISSSTE, Mexico City, MEX; 2 Department of Ophthalmic Pathology, Asociación para Evitar la Ceguera en México, I.A.P., Mexico City, MEX; 3 Department of Internal Medicine, Centro Médico Nacional 20 de Noviembre, Mexico City, MEX

**Keywords:** extra-medullary plasmacytoma, immuno, ocular tumor, radiotherapy (rt), surgical resection

## Abstract

This case report presents an extremely rare clinical case of a patient who presented with visual impairment, eye pain, and perforation. In this situation, an enucleation of the affected eye was performed, followed by a biopsy, which allowed the diagnosis to be established. From there, a multidisciplinary study was conducted to follow up and implement a definitive treatment.

## Introduction

Plasmacytomas are plasma cell neoplasms classified into two main types: solitary bone plasmacytomas (SBPs) and extramedullary plasmacytomas (EMPs). These entities differ from multiple myeloma due to the absence of the defining diagnostic criteria of the latter, such as normal hematological parameters (including reticulocytes), an unremarkable bone marrow biopsy, and normal immunoglobulin levels. Additionally, plasmacytomas are characterized by the isolated location of the lesion in a specific organ or structure of the body. The most common sites of incidence are the digestive system, followed by the respiratory system, with rates of 0.063/100,000 in women and 0.078/100,000 in men [1]. Currently, to our knowledge, no cases have been reported involving the entire eyeball, making this case particularly interesting.

This case report presents a male patient admitted to the Ophthalmology Department with vision loss and ocular pain, secondary to ocular perforation. This was the initial manifestation of his condition. The enucleation of the eyeball was performed for histopathological study. Histopathological and immunohistochemical analyses of the specimen confirmed the diagnosis of plasmacytoma. Given this finding, it was necessary to rule out systemic involvement, and the Hematology Department provided support to exclude the presence of multiple myeloma. Multiple myeloma was ruled out, and radiotherapy (RT) was initiated as the standard treatment.

It is important to note that, while progression to multiple myeloma is common in cases of SBPs, patients with EMPs generally have a better long-term prognosis. This case highlights the importance of a multidisciplinary approach for timely diagnosis and treatment.

In the following sections, key aspects related to diagnosis, treatment, prognosis, incidence, and immunohistochemical markers used in similar cases are reviewed. Additionally, advancements in emerging therapies and the factors influencing therapeutic decisions in this context are discussed.

## Case presentation

A 64-year-old man with a history of systemic arterial hypertension, currently on treatment with losartan 50 mg every 24 hours, and without any family history relevant to the current condition, presents with a clinical picture characterized by a weight loss of 10 kg over the course of one month, accompanied by eye pain, redness, and decreased vision in the right eye, prompting him to seek evaluation in a hospital setting.

On general examination, the patient was ectomorphic in build, with a facial expression indicative of pain. Cranial nerves were intact, and there were no palpable nodules. The rest of the examination was unremarkable.

**Figure 1:**
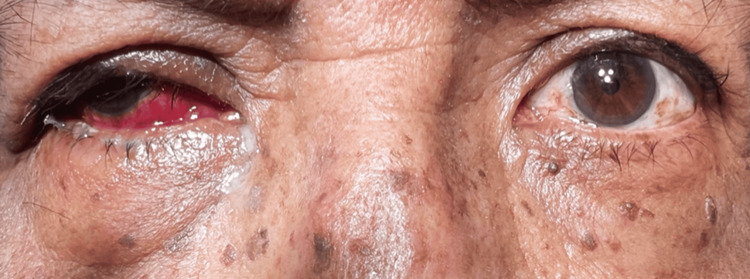
Sensory exotropia

**Figure 2:**
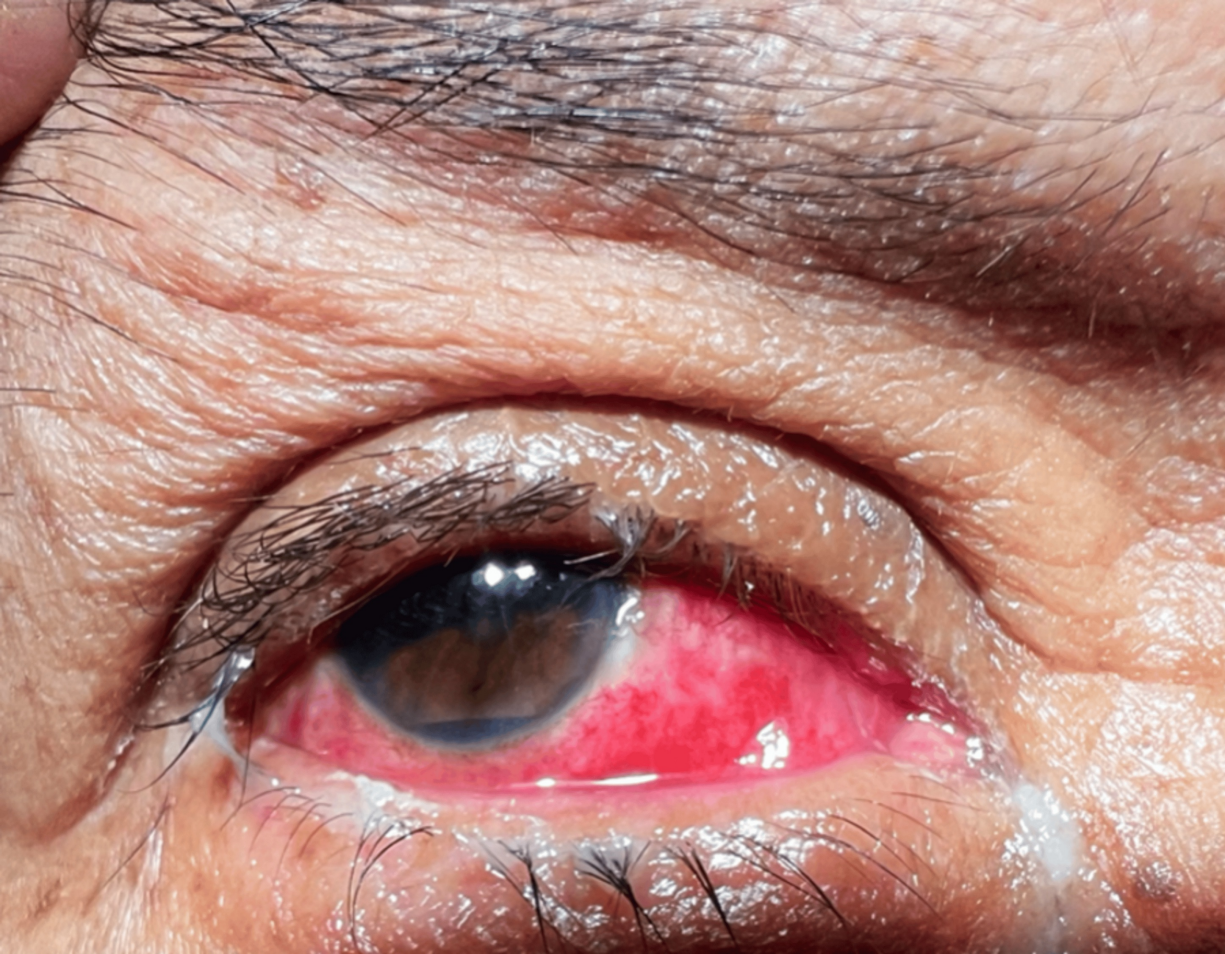
Anterior segment

Ophthalmologic examination revealed facial asymmetry, moderate ptosis (3 mm), sensory exotropia of the right eye, and abundant conjunctival discharge (Table 1; Figure 1). The anterior chamber was formed with hypopyon, hematic remains, 360º rubeosis iridis, and pupillary seclusion (Figure 2).

**Table 1:** Ophthalmic examination

Examination	Right eye	Left eye
Visual acuity	No light perception	20/60 (uncorrected), 20/25 (best corrected)
Eye movements	-2 limitation to adduction, -2 limitation to supralevoduction and infralevoduction	No limitation
Pupilar reflexes	Absent	No alterations
Intraocular pressure	20 mmHg	13 mmHg

**Figure 3:**
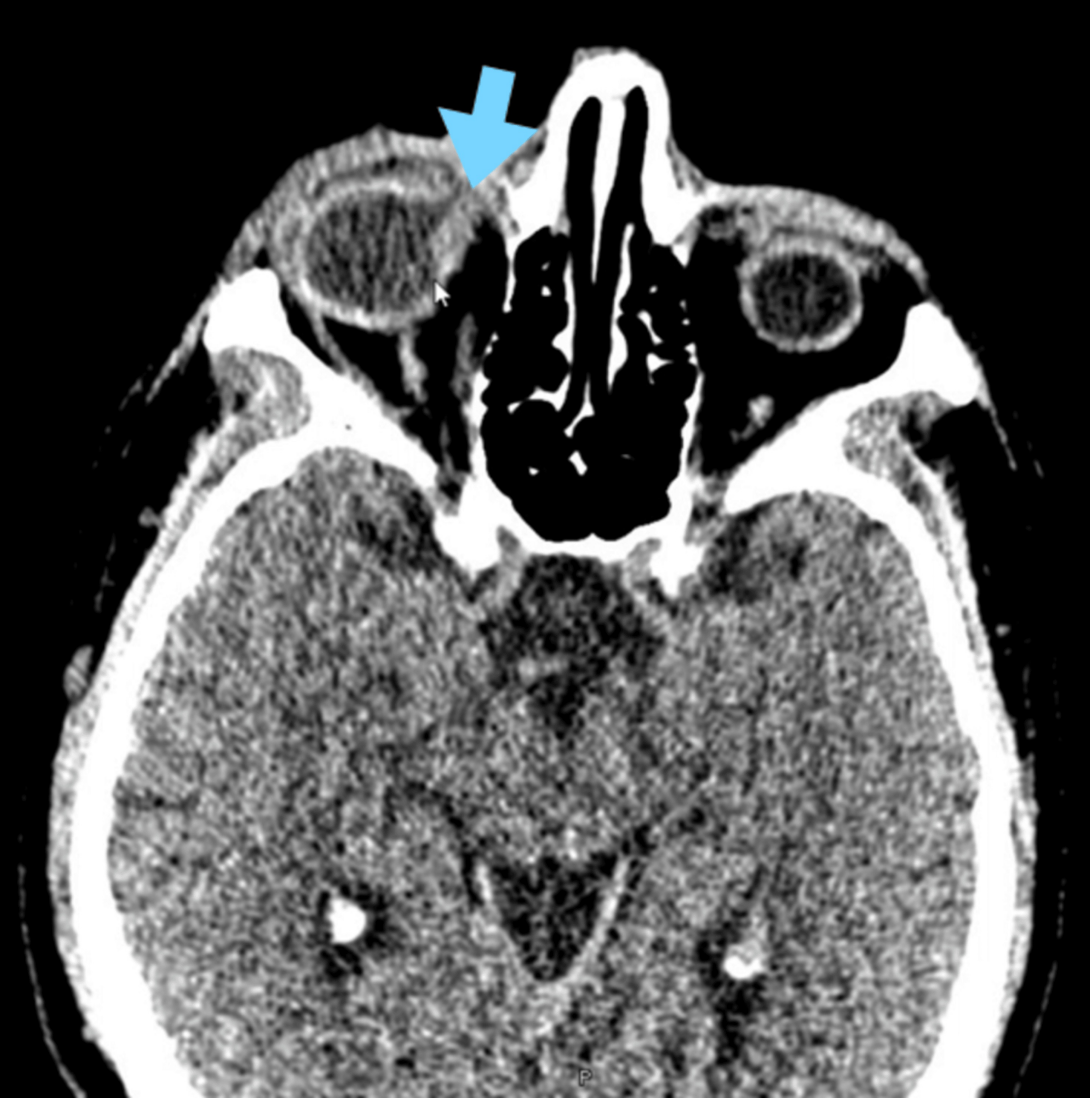
CT scan A vitreous-conjunctival fistula is observed at the arrow. CT: computed tomography

A high-resolution computed tomography (CT) scan of the orbits and paranasal sinuses revealed the following findings: soft tissue edema and vitreous-conjunctival fistula of the right eye (Figure 3).

**Figure 4:**
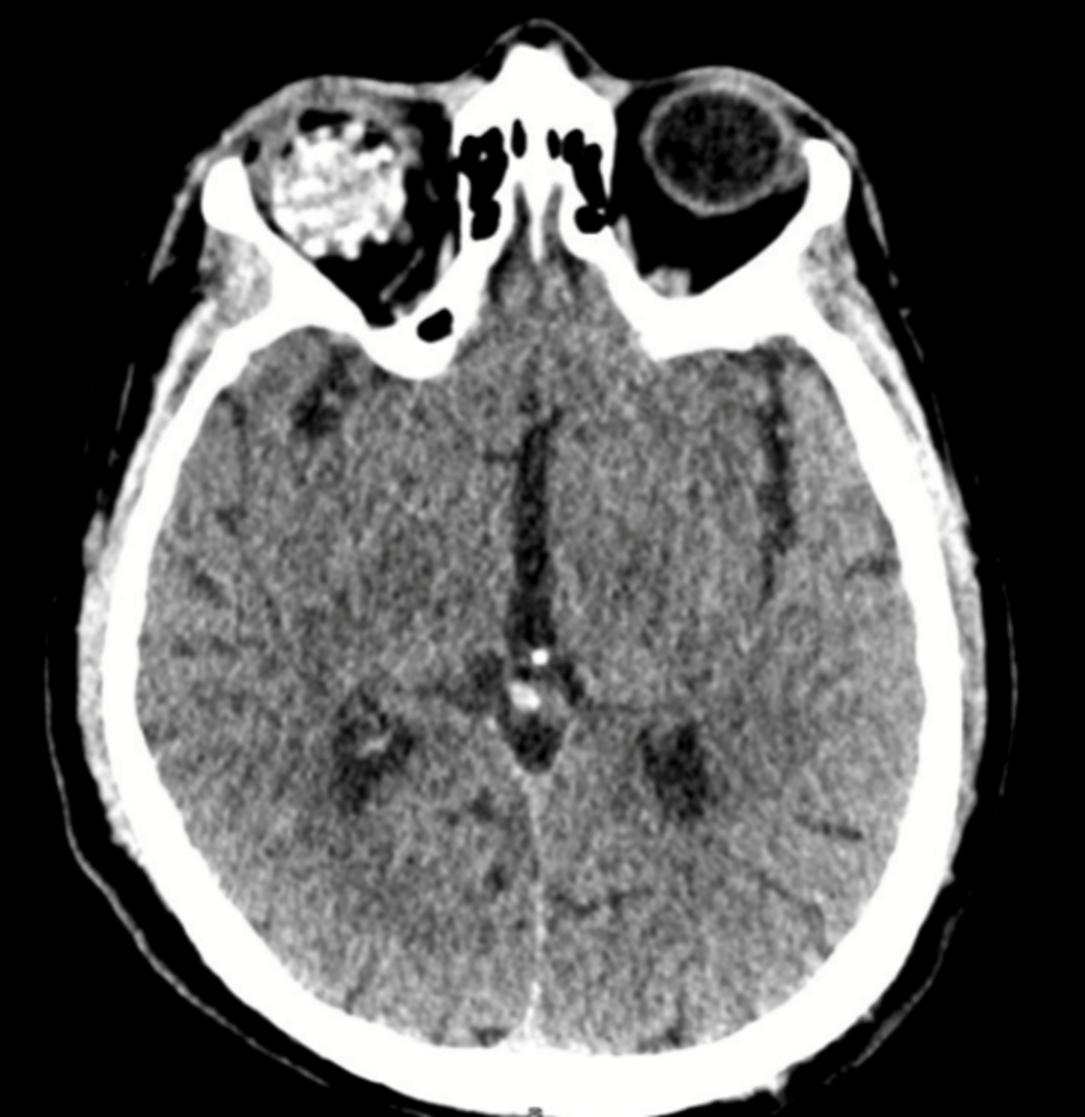
CT scan The axial section shows the hyperdense multi-celled implant. CT: computed tomography

Due to ocular perforation demonstrated on tomography and a painful, blind eye, enucleation of the right eye was decided upon. During this procedure, a 24 mm multi-celled spherical implant made of polylactic acid was placed (Figure 4). An autograft of abdominal fat tissue was performed.

**Figure 5:**
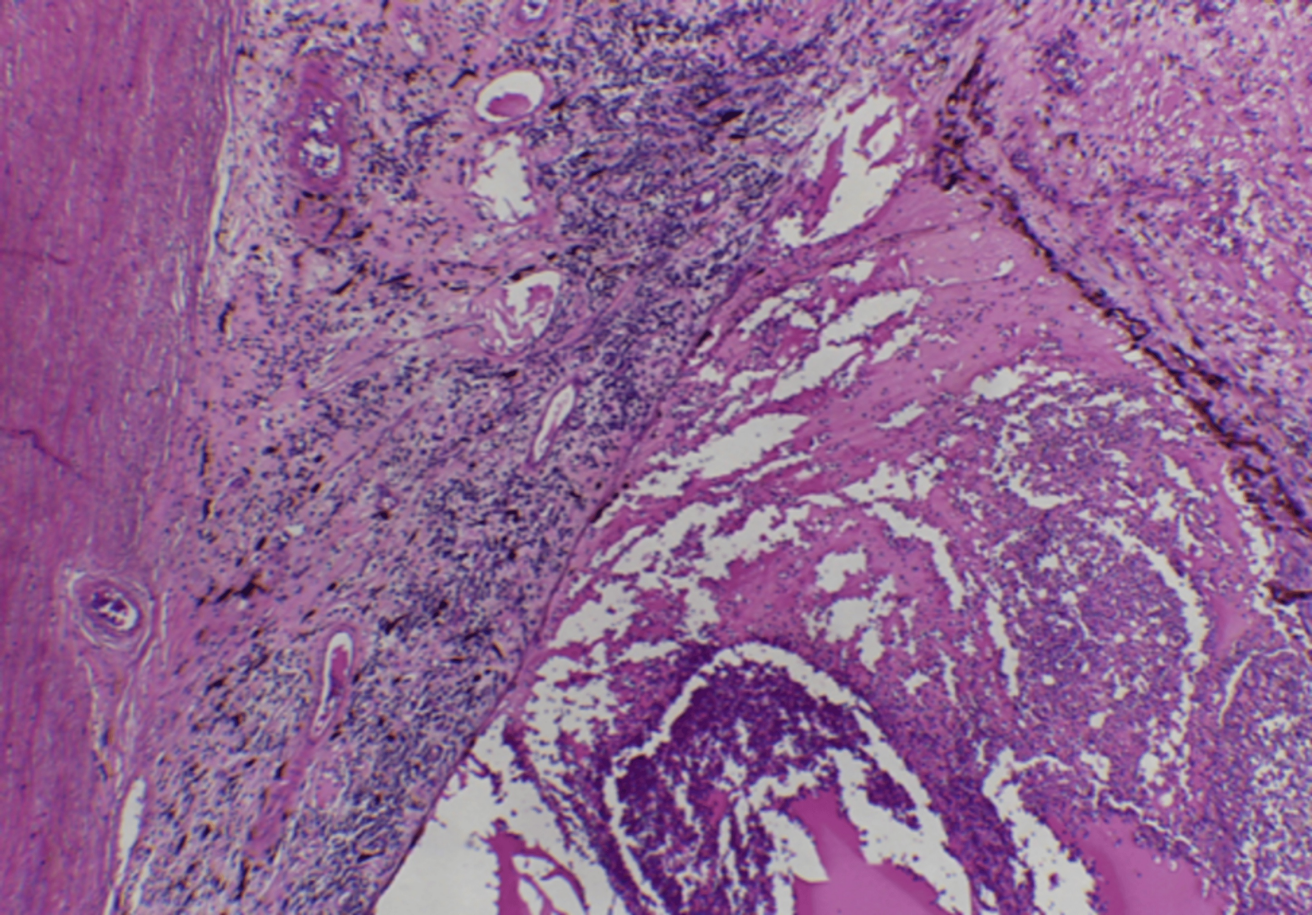
Photomicrograph (H&E, 4x) Photomicrograph showing widening of the uveal tract stroma, as well as the vitreous chamber, both occupied by a diffuse lymphoplasmacytic infiltrate. H&E: hematoxylin and eosin

**Figure 6:**
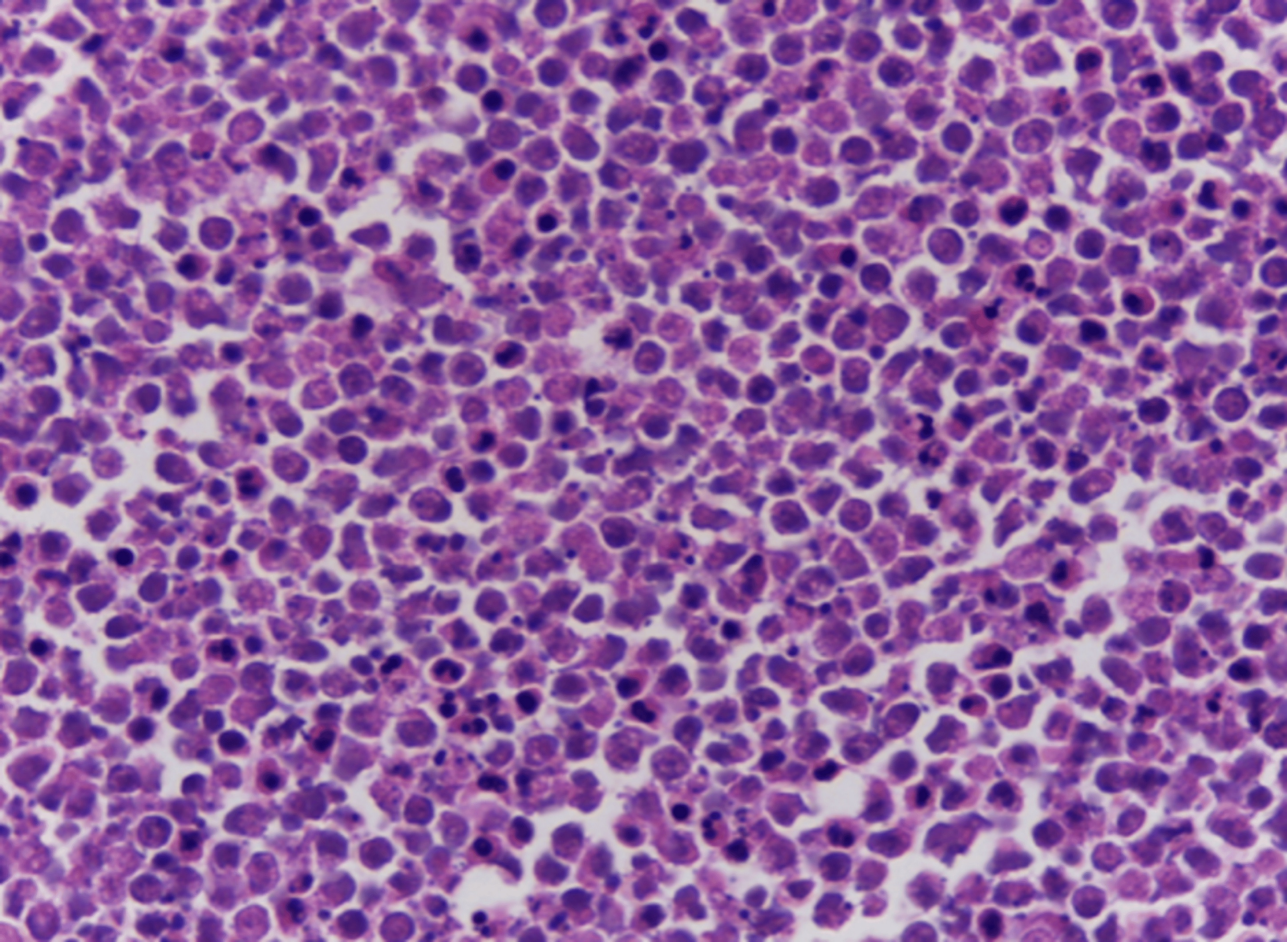
Photomicrograph (H&E, 40x) Photomicrograph showing a diffuse infiltrate of medium-sized plasma cells, many of which have apoptotic changes. H&E: hematoxylin and eosin

The specimen was sent for histopathological examination, revealing the following findings: microscopic examination showed a marked thickening of the stroma of the uveal tract, including the pars plana and pars plicata of the ciliary body and the entire choroid, due to a dense infiltrate of lymphocytes and plasma cells mixed with amorphous eosinophilic exudate and recent hemorrhage. Immunohistochemistry analysis revealed the presence of a plasma cell neoplasm (CD138+ and CD56+). A light chain restriction was observed, with a negative result for kappa and a positive result for lambda (Figures 5-6).

The patient was referred to the hematology service to continue the protocol, where various complementary studies were carried out to confirm the diagnosis of multiple myeloma. The results indicated a bone marrow biopsy with no evidence of neoplastic infiltration, normal serum calcium levels, cerebrospinal fluid (CSF) samples within normal parameters, and an unremarkable blood smear. In addition, serum immunoglobulins and reticulocytes showed no abnormalities. Based on these findings, the diagnosis of extramedullary ocular solitary plasmacytoma was established (Tables 2-3).

**Table 2:** Cerebrospinal fluid examination

Characteristics	Results
Color	Colorless
Appearance	Transparent
White blood cell (WBC)	3 per mm^3^
Red blood cell (RBC)	2 per mm^3^
Glucose	53 mg/dL
Proteins	40.0 mg/dL
pH	7.62
India ink	Negative
Lactate dehydrogenase (LDH)	30 u/L
Lactate	25.2 mg/dL

**Table 3:** Laboratory results

Test	Result (mg/dL)	Normal range (mg/dL)
IgA	407	<645
IgE	31.8	<100
IgG	1670	540-1822
IgM	197	22-240
Hemoglobin	15.5	17.00
Reticulocytes	1.77%	<2%
White blood cells	4.48 x 10^3^/mcL	4.5-10 x 10^3^/mcL
Calcium	9.4	8.2-10.2

**Figure 7:**
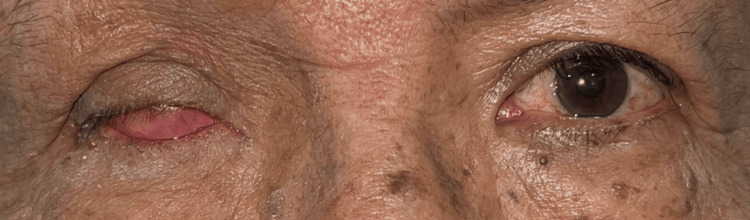
Postoperative photograph Patient with a fenestrated acrylic shell conformer.

After diagnosis, the patient was referred to the radio-oncology service to begin treatment with RT. A total of 20 sessions of RT were performed, with a total dose of 40 Gy. Six months after treatment, the orbital cavity was reconstructed, including the reformation of the sac fundus (Figure 7).

## Discussion

Plasmacytomas are plasma dyscrasias that derive from mature B cells. These tumors are a rare form of plasmocellular neoplasms, including SBPs and EMPs. SBPs are mainly located in the axial skeleton, commonly affecting the spine, ribs, and pelvis, while EMPs usually involve soft tissues, especially in the head and neck region [1,2].

The incidence of plasmacytomas is relatively low compared to other hematologic malignancies. It is estimated that plasmacytomas account for about 3%-5% of all plasma cell dyscrasias [3]. Approximately 70% of the cases correspond to SBPs, while the remaining 30% are EMPs [3]. The predicted incidence of EMPs is extremely low, with 0.063/100,000 in women and 0.078/100,000 in men. Plasmacytomas tend to occur in people over 50, with a peak incidence in the sixth decade of life. A higher prevalence is observed in men, with a male-to-female ratio of approximately 2:1 [4].

Epidemiology varies slightly across geographic regions and ethnic groups, but most studies agree that patients of European descent have a higher rate of plasmacytomas compared to those of African or Asian descent.

Diagnosis

Diagnostic Criteria

**Figure 8:**
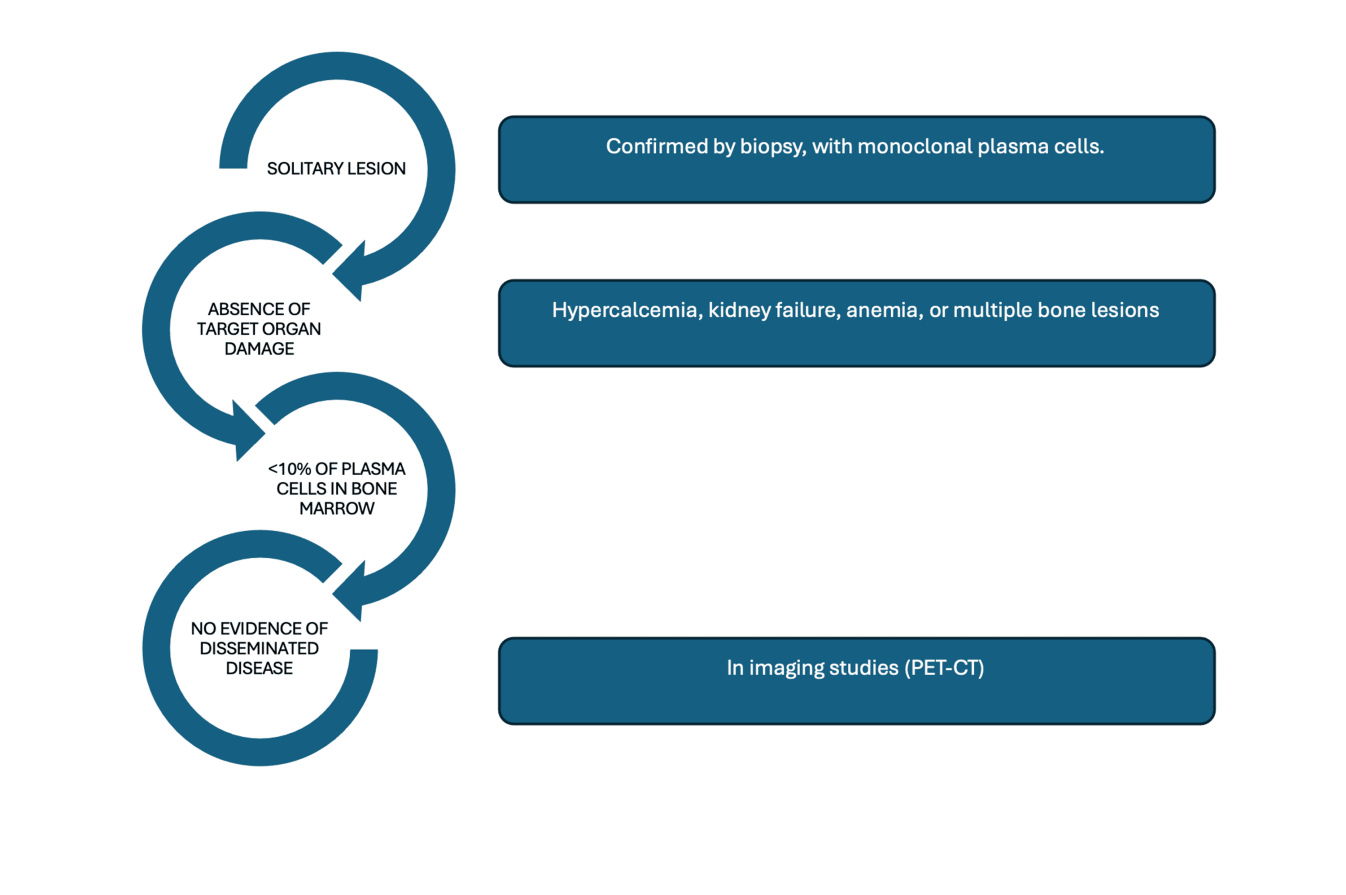
Diagnostic criteria PET-CT: positron emission tomography-computed tomography

To differentiate plasmacytomas from multiple myeloma and establish an accurate diagnosis, the following criteria must be met, as shown in Figure 8 [5-10].

Diagnostic Imaging

The diagnosis of SBPs and EMPs is based on advanced imaging, biopsies, and laboratory studies. For EMPs, MRI is crucial to evaluate soft tissue infiltration. FDG 18F positron emission tomography-computed tomography (PET-CT) has become a key tool for detecting disease extent and differentiating it from multiple myeloma [5,11].

Diagnosis by Immunohistochemistry

Immunohistochemistry is essential to confirm the diagnosis and characterize plasmacytomas. In biopsies, plasmacytomas show a proliferation of monoclonal plasma cells. The main immunohistochemical markers used include: (1) CD20: although plasma cells do not generally express CD20, a small subgroup of plasmacytomas may be positive for this marker, which may influence treatment, particularly with targeted therapies such as rituximab [8]; (2) CD79a: antibody labels are the most reliable way to detect B-cells in paraffin-embedded samples of precursor B-cell acute lymphoblastic leukemia; (3) Ki-67: it is a proliferation marker that provides information about the mitotic activity of tumor cells. A high proliferation index in Ki-67 suggests a more aggressive behavior of the tumor; (4) CD138: this is the most common marker for identifying plasma cells; (5) CD56: it is a marker frequently observed in malignant plasma cells, and its expression is associated with greater aggressiveness and a worse prognosis.

Treatment

Throughout the disease, treatment decisions are influenced by factors such as age, overall health, existing medical conditions, and suitability for stem cell transplantation [12]. Treatment of EMPs varies depending on the location of the tumor and its extent, but RT is considered the primary therapeutic modality for both entities. Recommended doses range from 40 to 60 Gy, and RT has been shown to provide excellent local control, with control rates of 80%-100%, depending on the dose administered and the lesion size. Specifically, delivering more than 40 Gy can lead to a higher rate of complete remission and a reduced likelihood of local relapse. Higher radiation doses are more effective in eradicating cancer cells within the localized tumor, thereby improving the chances of complete disease control in the treated area [5].

Surgery is a secondary therapeutic option, indicated primarily for patients with injuries that cause mechanical complications, such as pathological fractures, or for those with extramedullary tumors that cause obstruction or compression of adjacent structures, such as the airways. Surgical resection is also considered in situations where the tumor does not respond to RT or in cases of recurrence [1].

New therapies and future perspectives

New therapeutic strategies are being developed to improve the management of plasmacytomas and reduce the rate of progression to multiple myeloma. Bisphosphonates, such as zoledronic acid, have shown potential in preventing related bone events and inhibiting the progression of SBPs to multiple myeloma [13]. In addition, proteasome inhibitors, such as bortezomib, have shown promise in preclinical studies and patients with refractory or recurrent plasmacytomas [9,14].

Prognosis

The prognosis of plasmacytomas varies depending on the type and management of the patient. In general, patients with SBPs have a higher chance of progressing to multiple myeloma compared to those with EMPs. The rate of progression in EMPs is much lower, around 10%-30% over 10 years [2-15]. CD79a reacted with the vast majority (more than 95%) of B-cell neoplasms, covering all stages of B-cell maturation [16]. CD56 is a marker commonly found in malignant plasma cells, and its presence is linked to increased aggressiveness and poorer prognosis [17].

Factors that predict a worse prognosis include the persistence of monoclonal protein in serum after RT, the identification of more than one lesion on advanced imaging studies, residual plasma cells in the bone marrow, and tumor sizes >5 cm. In addition, PET-CT has become a valuable tool for follow-up, as it can identify disease relapse before clinical symptoms appear [15].

## Conclusions

Plasmacytomas are rare neoplasms commonly located in bone tissue, as well as in the gastrointestinal and respiratory systems, but this case is particularly unusual due to the lesion involving the entire eyeball with a nonspecific presentation. Histopathological examination plays a key role in achieving an accurate diagnosis, and systemic involvement must be ruled out through tests such as bone marrow biopsy, serum calcium levels, reticulocyte count, and immunoglobulin testing, to confirm the diagnosis of solitary plasmacytoma.

Treatment typically includes surgery or RT, which is the first-line approach and achieves a response in over 90% of solitary lesions. Although progression to multiple myeloma occurs in approximately 30% of patients, close monitoring and a multidisciplinary approach are essential for effective disease management and early detection of systemic progression, and chemotherapy is reserved for specific cases where additional intervention is needed.
